# Enhancement of Green Tires Performance through Ultrasound-Assisted Mixing

**DOI:** 10.3390/polym14030418

**Published:** 2022-01-20

**Authors:** Yaohua Cheng, Qianting Wang

**Affiliations:** 1 School of Materials Science and Engineering, Fuzhou University, Fuzhou 350108, China; qustcyh2013@126.com; 2 School of Materials Science and Engineering, Fujian University of Technology, Fuzhou 350118, China

**Keywords:** green tires, ultrasonic wave, devil’s triangle, mixing

## Abstract

Combined with the traditional internal mixing process, a custom-built ultrasonic generator was introduced in this study. The effect of ultrasonic parameters on the comprehensive performance of tread rubber formulations was investigated. Compared to the traditional mixing process without ultrasonic wave loading, the introduction of ultrasonic enhanced the dispersion and distribution of composite particles in the rubber matrix and improved the overall performance of rubber products. The devil’s triangle relationship among the rolling resistance, wet skid resistance, and abrasion resistance of tires was improved. When the wet skid resistance was slightly lost, the rolling resistance and wear rate were effectively reduced. This study provides new insights into a strategy for optimizing the mixing process of the traditional internal mixer, reducing vehicle emissions, extending the service life of tires, and promoting the development of green tires.

## 1. Introduction

Today, cars have become an indispensable means of transportation for every family. With the increasingly tight global energy supply and the gradual deterioration of the ecological environment, the pollution problems caused by automobile emissions and waste tires have become increasingly prominent [1,2,3,4,5,6,7]. Only by promoting green tires that have low rolling resistance, high wear resistance, and excellent comprehensive performance can we cope with the above problems [8,9]. Therefore, the EU introduced a tire countermark law in 2009, and the Green Tire Technology Specification was published by the China Rubber Industry Association in 2014. Premier Keqiang Li set clear goals for the work of optimizing the industrial and energy mix in his government work report in March 2021. The proposal of these policies showed that green tires are the direction of future development.

To prepare green tires with excellent performance, new materials, mixing technologies, and process combinations have been continuously developed and innovated in recent years. Unfortunately, most of them have some problems, which make them not yet suitable for industrialization. For example, the application of graphene and carbon nanotubes in rubber formulations can improve the tensile strength, tear resistance, wear resistance, electrical conductivity, thermal conductivity, and other properties of rubber, but the production cost of rubber will be greatly increased [10,11,12,13,14]. The low-temperature primary mixing process that uses multiple open mixers and a single internal mixer for supplementary mixing has been promoted for a small range of traditional mixing method applications [15]. However, the application of multiple open mills not only increases the initial investment cost of the factory but also produces environmental pollution.

Although the application of the tandem internal mixer improves the processing quality and production efficiency of rubber, the entire control system involving two internal mixers is more complicated, and the rotor processing cost is high, which greatly increases the production cost. In terms of advanced continuous mixing, the continuity of the rubber mixing process has been achieved to a certain extent. However, in the early stage of mixing, it is necessary to refine and atomize the various materials of the formula, and the ratio among them is difficult to accurately control, which makes it challenging to guarantee the quality of rubber. Recently, these problems have been solved by the organic combination of an internal mixer, double-cone feeder, and twin-screw mixer by Wang and colleagues [16,17,18,19]. However, many experiments are required to determine the accurate parameter combination in the early stages of production to achieve coordinated work and precise control between multiple devices, which requires a large financial investment.

Therefore, severe challenges to the improvement in the traditional internal mixer and the relevant process are posed at the current stage.

Ultrasonic waves have been recognized as an important means for improving the properties of polymer materials in the past few years. Price [20] found they could reduce the molecular weight of the polymer solution and make it a stable value. This result demonstrated that it is possible to control the molecular weight of the polymer by ultrasonic waves. Ultrasonic waves have been used for the extrusion of polymeric materials, desulfurization of rubber [21,22], plastic decrosslinking [23], and compatibilization of polymer blends [24,25,26]. In addition, the ultrasonic waves could also increase the storage modulus, tensile strength, and dispersion of internal dispersed phases of composite materials [27,28]. With the increase in the use of wet rubber mixing, ultrasonic waves are often used in the early stages of pretreatment of natural rubber latex and the preparation of master rubber [29,30,31,32].

In this study, ultrasonic waves were introduced into the traditional internal mixer mixing process to explore the influence of different ultrasonic parameters on the performance of tread rubber formulations, seeking the best process parameters for preparing green tire products with good dispersibility and distribution. This provides a guide for optimizing the mixing process of the traditional internal mixer.

## 2. Experimental Section

### 2.1. Experimental Equipment and Ultrasonic Loading Method

**Figure 1:**
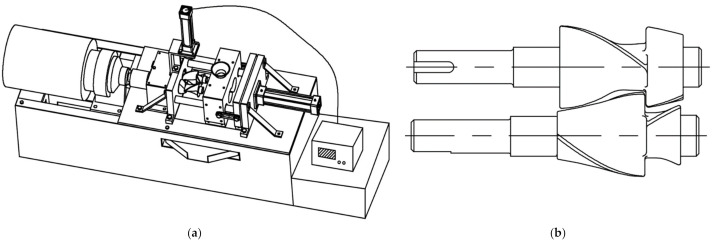
(**a**) The experimental equipment; (**b**) the rotor configuration.

The experimental equipment (Qingdao Yinglang Rubber Equipment Co., Ltd, Qingdao, China) and its rotor configuration are shown in Figure 1. The maximum working volume of the experimental equipment was 0.3 L.

**Figure 2:**
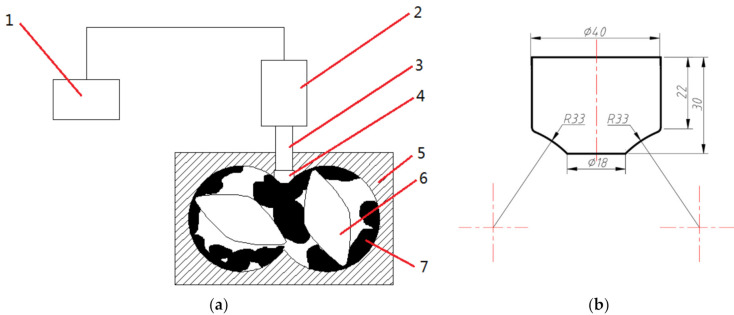
The custom-built ultrasonic generator and tool hand: (**a**) 1, Ultrasonic generator; 2, transducer; 3, amplitude; 4, tool head; 5, mixing room; 6, rotor; 7, rubber; (**b**) The specific size of the tool head.

The custom-built ultrasonic generator is shown in Figure 2.

The custom-built ultrasonic generator is composed of 1, 2, 3, and 4 in Figure 2, which replace the upper top mechanism of the internal mixer. The ultrasonic wave was loaded by direct contact between the tool head (4) and the rubber during the experiment.

### 2.2. Materials

Solution-polymerized styrene butadiene rubber (SSBR), cis-polybutadiene (BR), stearic acid and ZnO were purchased from Sinopec Group (Beijing, China); *N*-1,3-dimethylbutyl-*N*’-phenyl-p-phenylenediamine (DMPPD), Accelerator CZ (*N*-cyclohexylbenzothiazole-2-sulphenamide) and sulfur were purchased from Shandong Shangshun Chemical Co., Ltd. (Heze, China); silica and Si69 were purchased from Solvay (Brussels, Belgium); CB N234 was obtained from Cabot Corporation (Boston, MA, USA); and aromatic oil was obtained from Hansen & Rosenthal (Hamburg, Germany).

### 2.3. Experimental Formula

The rubber formula used in this study is described in Table 1.

### 2.4. Experimental Method

Table 2 shows the experimental scheme. The mixing process was as follows: the filling coefficient of the mixer was 65%, the cooling water temperature was 40 °C, and the rotating speed as 65 r/min.

In the mixing process, the corresponding periods of ultrasonic waves of different powers were as shown in Table 3.

An open mill was used to add the curing system to the mixture, which was then vulcanized by a flat plate vulcanizing machine. Various performance tests were carried out and the main test items were as follows: the Mooney viscosity values of the rubber compounds were evaluated using a Mooney viscometer (UM-2050, GOTECH Testing Machines Inc., Ltd., Taichung, Taiwan) according to ISO 289-2: 2016. The tensile strength and tear strength were measured using a U-CAN TS2005b universal testing machine (TS 2005 b, GOTECH Testing Machines Inc., Ltd., Taichung, Taiwan) according to ASTM D412 and ASTM D 624. RPA2000 was used to test the dynamic rheological properties. Strain scanning of the mixing rubber was performed with the following parameters: frequency 1 Hz, temperature 60 °C, and strain range 0.1 °C. The dynamic strain was measured by dynamic mechanical analysis (DMA; GABOMETER-150, GABO, München, Germany). The tensile mode was used in temperature scanning; the frequency was 10 Hz, and the heating rate was 2 °C/min. DIN abrasion was tested using a GT-2012-D DIN abrasion machine according to the GB/T 9867-2008 standard. Filler dispersion was tested using a DisperGRADER (Alpha Company, Bellingham, WA, USA) dispersion tester, which automatically obtained sample dispersion ratings according to ISO11345 and ASTM D7723 standards. The cross-section of the sample after tensile fracture, which adhered to the conductive adhesive and was fixed on the sample table for gold spraying, was observed under a scanning electron microscope (SU8000, Hitachi, Japan). The crosslink density values were determined by the equilibrium swelling method using toluene as the solvent.

## 3. Results and Discussion

The effects of different ultrasonic parameters on rubber properties are shown in Table 4.

### 3.1. The Effects of Different Ultrasonic Parameters on the Properties of Vulcanizates

**Figure 3:**
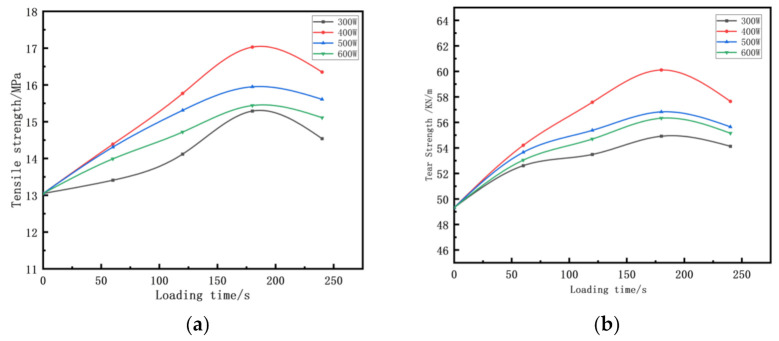
Effect of different ultrasonic parameters on the mechanical properties of vulcanizate: (**a**) tensile strength and (**b**) tear strength.

Figure 3 shows the effect of different ultrasonic parameters on the mechanical properties of vulcanizates. It can be seen from Figure 3 that the mechanical properties of the vulcanized rubber were lower under the condition of not loading the ultrasonic wave.

The matrix glue and compounding agent particles were mainly sheared and broken under the action of shear stress in the traditional internal mixing process. The physical and mechanical properties of the final specimens were average. This is because the different properties of the various compounding agent particles in the formulation made it difficult for them to be uniformly dispersed under the action of shear stress. For example, the silica particles have high polarity, high surface energy, and easily agglomerate, so they easily adsorb other compounding agent particles and form agglomerates in the rubber during the mixing process. It also harms the silane coupling reaction between the silica and the silane coupling agent.

With the introduction of ultrasonic waves, the mechanical properties of vulcanizates first increased and then decreased. When the ultrasonic wave parameters were 400 W and 180 s, the compound had the best performance. The tensile strength increased by 28.72% and the tear strength increased by 21.85%.

The main reasons for these findings are as follows:

(1) The impact force due to the introduction of ultrasonic waves forms a synergistic effect with the shear stress, which accelerates the sliding and shedding speed of the compound particles in the mixing process, promoting their relative flow in the rubber matrix, and improving their dispersion effect;

(2) During the mixing process, the bubbles formed by the moisture and air inside the rubber matrix are continuously compressed and ruptured under the action of ultrasonic vibration, which is equivalent to introducing countless tiny and irregular extensional and extensional flows. The introduction of the new contact area produces more than the shear flow and promotes the progress of decentralized mixing;

(3) Under the combined action of tensile stress and alternating strong and weak shear stress, the increased rate of the contact interface between the compounding agent particles and the rubber matrix is also improved to a certain extent, which enhances the effect of distributed mixing;

(4) The silane coupling reaction between silica and Si69 improves with the increase in the dispersion and distribution of the compound particles, which improves the mechanical properties of vulcanized rubber;

(5) As the ultrasonic loading time reaches 240 s, although the dispersed compounding agent particles are newly agglomerated inside the rubber matrix, the mechanical properties of the vulcanizate are reduced, but it is still better than the style prepared without ultrasonic waves.

ML and MH represent the minimum torque and maximum torque, respectively. MH and ML can reflect the size of the crosslinking density of the rubber: the greater the difference, the higher the crosslinking density [33]. As shown in Table 5, the ultrasonic wave significantly improved the crosslinking density of rubber, and reached the maximum when the ultrasonic parameters were 400 W and 180 s. This is because the ultrasonic wave enhanced the distribution and dispersion effect of compounding agent particles in the rubber, and increased the contact area between compounding agent particles and the rubber matrix in the entire system, thereby enhancing the crosslinking density of the rubber.

Above all, the introduction of ultrasonic waves can improve the effect of rubber mixing, enhance the dispersion and distribution of compounding particles in the rubber, and increase the crosslinking density of the rubber, so it has a positive effect on the improvement of the mechanical properties of the vulcanizate. Table 6 shows the values reported in other studies for different methods without using ultrasonic waves to improve the performance of rubber in some similar systems.

Table 7 shows the three-dimensional morphology of the vulcanized rubber section, dispersion of carbon black, and SEM photos under different ultrasonic loading parameters.

**Figure 4:**
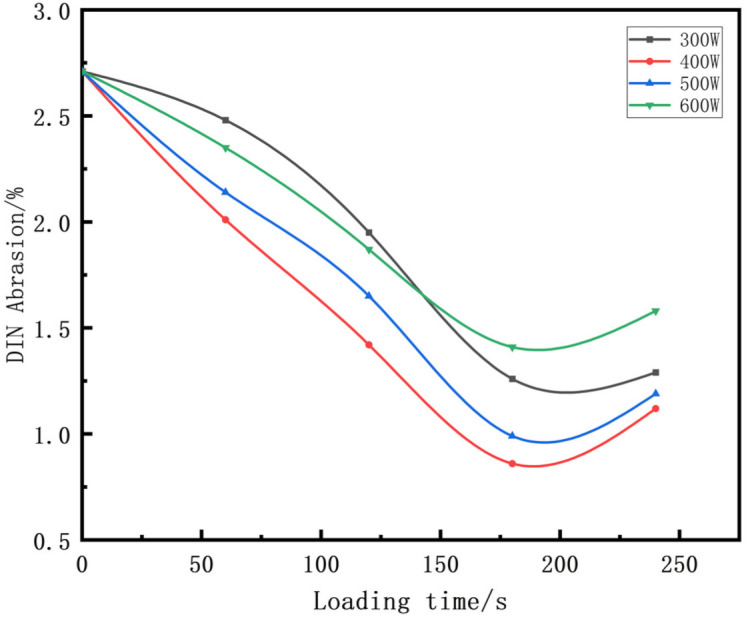
Effect of different ultrasonic parameters on the DIN abrasion of vulcanizates.

Table 7 shows that the vulcanizate cross-section was rough, the carbon black dispersion was poor, and there were many agglomerations when the ultrasonic waves were not loaded. The effect of dispersed mixing and distributed mixing was enhanced with the introduction of ultrasonic waves. The roughness of the vulcanized rubber section was gradually improved, the dispersion of carbon black was gradually increased, and the agglomeration phenomenon also gradually reduced. When the ultrasonic loading time reached 180 s, the optimal value was reached. As the ultrasonic loading time continued to accumulate, the dispersed compound particles had a secondary agglomeration in the rubber matrix, which adversely affected the morphology of the vulcanized rubber section and the dispersion of carbon black. This trend effectively echoes the previous analysis. The abrasion size of the vulcanized rubber and the dispersion and distribution effect of the compounding agent particles in the rubber matrix are inversely proportional. As shown in Figure 4, the vulcanized rubber wear curve also agrees with the above analysis.

### 3.2. The Effects of Different Ultrasonic Parameters on Mixing Rubber Mooney Viscosity

**Figure 5:**
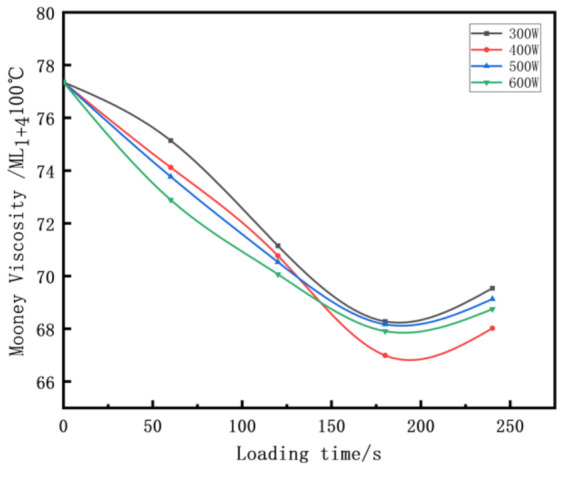
Effect of different ultrasonic parameters on mixing rubber Mooney viscosity.

The processability of a rubber compound is inversely proportional to the Mooney viscosity under certain conditions. Figure 5 shows that the Mooney viscosity of the rubber compound obtained under the mixing process conditions of the traditional internal mixer is relatively high.

This is because the rubber compound is mainly completed under the action of single shear stress in the traditional mixing process, which makes its Mooney viscosity high. The Mooney viscosity of the rubber compound tended to first decrease and then increase with the introduction of ultrasonic waves. When the ultrasonic parameters were 400 W and 180 s, the processability was optimal. This is because the rubber continuous expansion and compression time interval is much smaller than its own relaxation time under the action of ultrasonic waves. The rubber is subjected to multiple forces before returning to its original state, resulting in the accumulation of stress, which ultimately exacerbates the fracture of the rubber molecular chain segment, thereby reducing the Mooney viscosity [36]. With the continuous action of ultrasonic waves, the Mooney viscosity increased again, combined with the changing trend of the compounding agent particles in Table 7, which is because of the new agglomeration of the compounding agent particles that were dispersed [37].

### 3.3. The Effects of Different Ultrasonic Powers on the Processing Performance of Rubber

**Figure 6:**
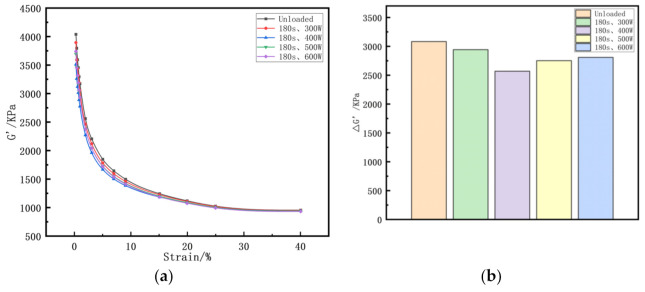
Rubber processing analyzer (RPA) curves of rubber with different ultrasonic powers (**a**)the G’ strain curves of rubber; (**b**) the Payne effect of rubber.

The Payne effect reflects the relationship between the deformation of a sample and the storage modulus. As shown in Figure 6a, with the help of RPA2000 (Alpha Company, Bellingham, WA, USA), the Payne effect of the samples was assessed by measuring the storage modulus (G’) when the strain increased from its lowest to its highest value (0.28–40%).

ΔG’ is used to characterize the Payne effect [33].

$$\Delta G’=G’\left(0.28\%\right)-G’\left(40\%\right)$$

Generally, the smaller the Payne effect, the more uniform the network structure formed by the components inside the sample, and the better the dispersion and distribution of the particles of each compounding agent. It can be seen from Figure 6b that the vibration, cavitation, and extensional flow caused by ultrasonic waves played a positive role in the mixing of rubber. When the ultrasonic parameters were 400 W and 180 s, the ΔG’ value was the smallest, and the compounding agent particles had the best dispersion and distribution in the rubber matrix.

### 3.4. The Effects of Different Ultrasonic Powers on Dynamic Mechanical Properties of Vulcanizates

Dynamic mechanical analysis refers to the mechanical response of composite materials under the action of alternating forces. Tan δ is the ratio of the loss modulus to the dynamic modulus.

**Figure 7:**
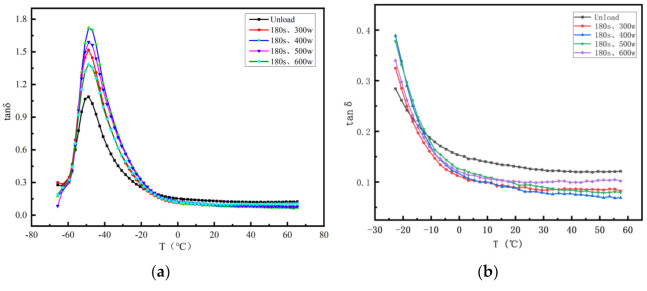
DMA curves of vulcanizates with different ultrasonic power: (**a**) the relationship between the loss factor (tan δ) and temperature (T) of the sample under different ultrasonic parameters, and (**b**) partially enlarged views.

When external force was applied to the sample, the larger the tan δ, the higher the in-ternal heat loss of the sample. It can be seen from Figure 7a that the ultrasonic wave enhanced the mixing effect of the rubber so that a more stable and uniform network structure was formed between the components inside the sample, which effectively limited the polymer chain segment sports. The performance was the best when the ultrasonic parameters were 400 W and 180 s.

In the tire industry, breaking through and improving the devil’s triangle relationship between the rolling resistance, wet skid resistance, and wear resistance of tires have always been of concern. When tan δ is 0 °C, it reflects the wet skid resistance of rubber: the larger the tan δ, the better the wet skid resistance. When tan δ is 60 °C, it reflects the rolling resistance of rubber: the smaller the tan δ, the smaller the rolling resistance. According to a comprehensive analysis of Figure 4 and Figure 7b, the introduction of ultrasound did not break the devil’s triangle relationship of tires, but it played a certain role in its improvement. Especially when the ultrasonic parameters were 400 W and 180 s, the rolling resistance was effectively reduced and the wear resistance was increased under the condition of slightly reducing the wet skid resistance.

## 4. Conclusions

In this work, ultrasonic waves were introduced into the traditional internal mixer process by a custom-built ultrasonic generator, and the effect of different ultrasonic parameters on the comprehensive performance of tread rubber formulation was studied. The results showed that:

(1) The ultrasonic waves can enhance the effects of dispersive mixing and distribute mixing, which have a positive effect on improving the comprehensiveness of rubber products.

(2) Compared to the traditional internal mixer process, when the ultrasonic parameter power was 400 W and the loading time was 180 s, the processability of the rubber product reached the best. At the same time, the tensile strength increased by 28.72%, and the tear strength increased by 21.85%.

(3) The ultrasonic waves can improve the relationship between the devil’s triangle factors in the tire industry. When the ultrasonic parameter power was 400 W and the loading time was 180 s, it effectively reduced the rolling resistance and improved the wear resistance while slightly reducing the wet slip resistance. The proposed method has certain advantages in production costs and process methods compared to that constructed by Wang et al. [16,17,18,19], which requires expensive continuous mixing equipment and complex process parameters;

(4) This method is important because it can help to reduce fuel consumption and vehicle emissions, and promote the development of green tires.

## Figures and Tables

**Table 1:** The formula of rubber tread adhesive.

Component	Formulation (phr)
SSBR	95
BR	32
ZnO	2
Stearic acid	2
DMPPD	2
Silica	45
Si69	5.4
CB N234	70
Aromatic oil	3
CZ	1.5
S	1.3

**Table 2:** Experimental scheme.

Experiment Number	Ultrasonic Power (W)	Loading Time (s)
Unloaded	0	0
1	300	60
2	300	120
3	300	180
4	300	240
5	400	60
6	400	120
7	400	180
8	400	240
9	500	60
10	500	120
11	500	180
12	500	240
13	600	60
14	600	120
15	600	180
16	600	240

**Table 3:** Mixing process.

Mixing Process						
1	Add rubber and mix for 30 s	Ultrasonic loading time (s)				
2	Add the additives and mix for 30 s	0	60	120	180	240
3	Add 1/2 silica and mix for 30 s					
4	Add carbon black and mix for 30 s		/			
5	Add the other 1/2 silica and mix for 30 s					
6	Add the oil and mix for 30 s			/		
7	Up and down the ram and mix for 30 s					
8	Up and down the ram and mix for 60 s				/	
9	Drop the rubber					

**Table 4:** Effect of different ultrasonic parameters on rubber properties.

Ultrasonic Power (W)	Loading Time (s)	Mooney Viscosity (ML_1+4_100 °C)	Tensile Strength (Mpa)	Tear Strength (KN/m)	DIN Abrasion (%)
0	0	77.35 ± 0.96	13.23 ± 0.94	49.33 ± 1.51	2.71 ± 0.091
300	60	75.14 ± 0.85	13.41 ± 0.86	52.61 ± 1.43	2.48 ± 0.063
300	120	71.15 ± 0.93	14.12 ± 0.92	53.49 ± 1.39	1.95 ± 0.068
300	180	68.28 ± 0.89	15.29 ± 0.89	54.92 ± 1.37	1.26 ± 0.066
300	240	69.54 ± 0.86	14.54 ± 0.90	54.13 ± 1.34	1.29 ± 0.060
400	60	74.12 ± 0.65	14.39 ± 0.88	54.21 ± 1.29	2.01 ± 0.075
400	120	70.77 ± 0.60	15.77 ± 0.89	57.58 ± 1.26	1.42 ± 0.069
400	180	66.99 ± 0.56	17.03 ± 0.86	60.11 ± 1.37	0.86 ± 0.071
400	240	68.02 ± 0.68	16.35 ± 0.91	57.65 ± 1.25	1.12 ± 0.072
500	60	73.77 ± 0.74	14.31 ± 0.71	53.66 ± 1.33	2.14 ± 0.083
500	120	70.53 ± 0.77	15.31 ± 0.78	55.38 ± 1.31	1.65 ± 0.084
500	180	68.17 ± 0.81	15.95 ± 0.76	56.83 ± 1.34	0.99 ± 0.079
500	240	69.13 ± 0.75	15.61 ± 0.77	55.65 ± 1.28	1.19 ± 0.085
600	60	72.89 ± 0.69	13.99 ± 0.78	53.05 ± 1.23	2.35 ± 0.074
600	120	70.07 ± 0.71	14.72 ± 0.81	54.70 ± 1.33	1.87 ± 0.069
600	180	67.91 ± 0.72	15.44 ± 0.86	56.33 ± 1.28	1.41 ± 0.073
600	240	68.75 ± 0.74	15.11 ± 0.85	55.15 ± 1.27	1.58 ± 0.072

**Table 5:** Processing properties of composite materials and the crosslinking density.

Test List	Ultrasonic Parameters: (1) Ultrasonic Power (W); (2) Loading Time (s)									
	(1)	(2)	(1)	(2)	(1)	(2)	(1)	(2)	(1)	(2)
	0	0	400	60	400	120	400	180	400	240
ML (dN·m)	3.48 ± 0.54		3.78 ± 0.43		3.54 ± 0.57		3.89 ± 0.45		3.92 ± 0.41	
MH (dN·m)	17.36 ± 0.35		17.75 ± 0.26		18.12 ± 0.31		18.83 ± 0.31		18.74 ± 0.19	
MH–ML (dN·m)	13.88 ± 0.19		13.97 ± 0.17		14.58 ± 0.26		14.94 ± 0.26		14.82 ± 0.22	
crosslinking density (mol·cm^−3^·10^−4^)	1.121		1.149		1.164		1.198		1.181	

**Table 6:** The previous references that reported other methods without using ultrasonic waves to improve the performance of rubber in some similar systems.

List	Tensile Strength	Tear Strength	Reference
1	↑10.20%	↑15.56%	[34]
2	↑4.60%	/	[35]
3	↑28.72%	↑21.85%	This Work

**Table 7:** Effect of different ultrasonic parameters on the microstructure of vulcanized rubber.

Ultrasonic Parameters		Three-Dimensional Morphology	Carbon Black Dispersion	SEM Photos
Ultrasonic Power (W)	Loading Time (s)			
0	0	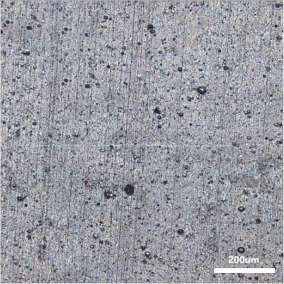	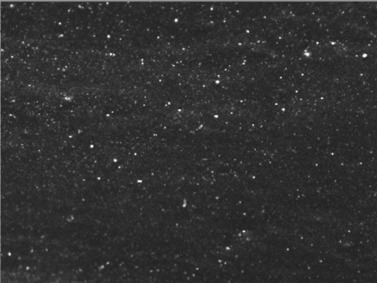	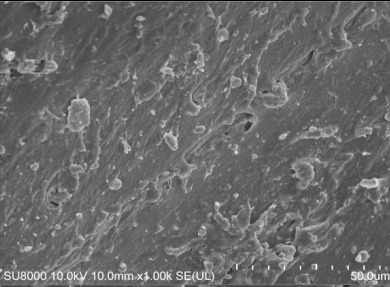
400	60	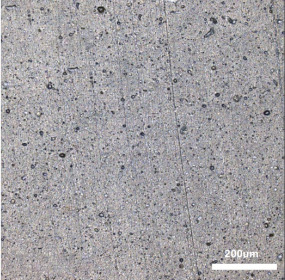	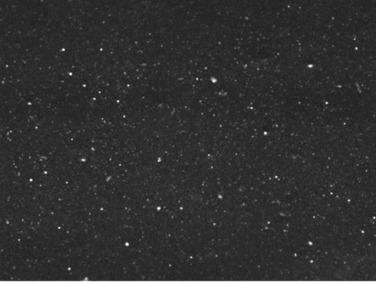	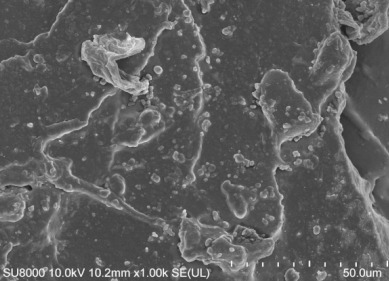
400	120	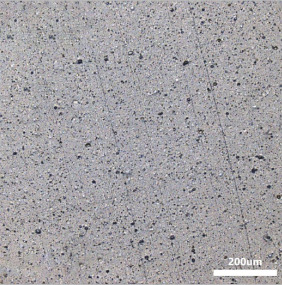	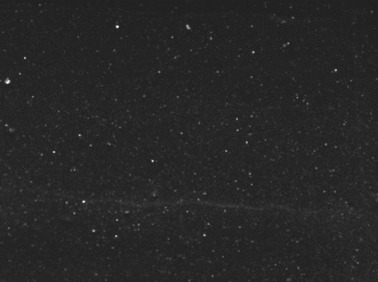	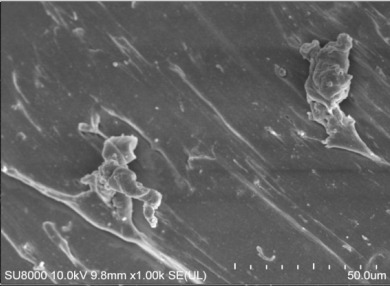
400	180	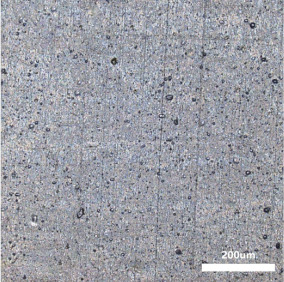	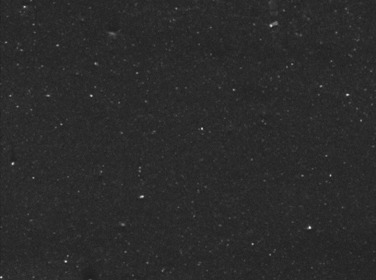	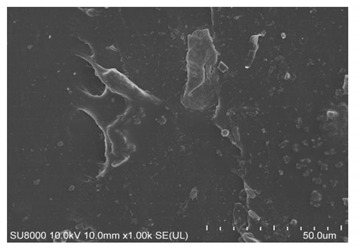
400	240	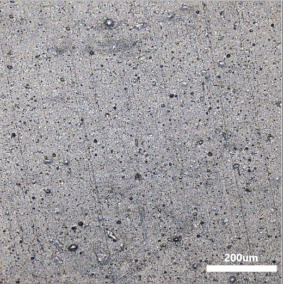	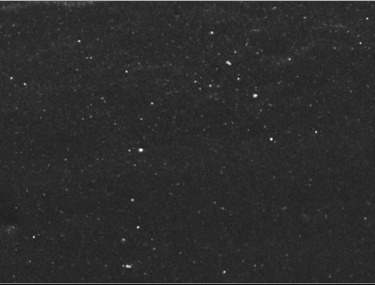	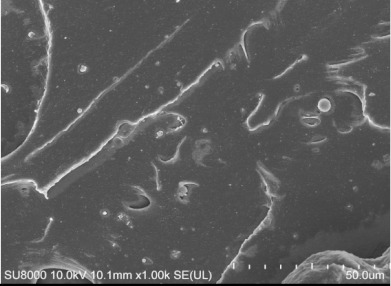

## Data Availability

The data presented in this study are available in this article and in [34,35].
